# Histological and Transmission Electron Microscopy Results after Embolization with HydroSoft/HydroFrame Coils in Experimental Swine Aneurysm

**DOI:** 10.1155/2019/4834535

**Published:** 2019-12-05

**Authors:** Seisuke Iseki, Yumiko Mitome-Mishima, Ikuko Ogino, Yasuo Suga, Kenji Yatomi, Senshu Nonaka, Nobukazu Miyamoto, Akihide Kondo, Munetaka Yamamoto, Hajime Arai, Hidenori Oishi

**Affiliations:** ^1^Department of Neurosurgery, Ota General Hospital, Kawasaki, Kanagawa 210-0024, Japan; ^2^Department of Neurosurgery, Tokyo Metropolitan Hiroo Hospital, Shibuya, Tokyo 150-0013, Japan; ^3^Department of Neurosurgery, Juntendo University School of Medicine, Bunkyo, Tokyo 113-8431, Japan; ^4^Department of Neurosurgery, Juntendo University Urayasu Hospital, Urayasu, Chiba 279-0021, Japan; ^5^Department of Neurology, Juntendo University School of Medicine, Bunkyo, Tokyo 113-8431, Japan; ^6^Department of Neuroendovascular Therapy, Juntendo University School of Medicine, Bunkyo, Tokyo 113-8431, Japan

## Abstract

Coiling and clipping are standard treatment strategies for cerebral aneurysms. Regardless of the strategy used, recanalization may affect the patient's prognosis. The aim of this study was to histologically and morphologically compare the tissue proliferation after coil embolization using bare platinum coils versus second-generation hydrogel coils (HydroSoft/HydroFrame; MicroVention, Inc., Aliso Viejo, CA, USA). Endothelial-like cell proliferation was seen in both groups at 2 weeks after surgery. Macroscopic findings showed a tighter layer at 4 weeks in the hydrogel coil group, and histological and immunohistochemical findings revealed endothelial cell proliferation. This layer became much thicker and tighter at 4 weeks after surgery. Aneurysms treated with second-generation hydrogel coils may be more stable and have a lower incidence of recanalization than those treated with bare platinum coils because of the tight endothelial layer proliferation.

## 1. Introduction

Rupture of a cerebral aneurysm causes subarachnoid hemorrhage, which readily leads to a severe clinical state. Surgical therapy (clipping) and endovascular therapy (coiling) are used to avoid cerebral aneurysm rupture. Since publication of the results of the International Subarachnoid Aneurysm Trial, endovascular treatment (coiling) has gained more importance in cerebral aneurysm treatment [1, 2]. Endovascular therapy has advanced through the development of various techniques such as the balloon remodeling technique and stent-assisted technique. Despite the advances in endovascular therapy, the curability of aneurysms remains lower than that obtained with clipping [3–6], especially for giant and wide-necked aneurysms, because of the higher recanalization rate after endovascular therapy than after clipping [7].

New-generation coils have been developed with polymer-platinum hybrid devices, degradable polymers, and expansible polymers. Matrix2 (Boston Scientific, Marlborough, MA, USA) is a degradable polymer device that was evaluated by Mitome-Mishima et al. [8] in a swine experimental aneurysm model. Expansible polymer devices have been developed because the increased volume of embolic devices within the aneurysm sac may provide better stability for thrombus organization [9].

Hydrogel coils consist of a platinum coil covered with an outer layer of hydrogel. The hydrogel material expands over a predetermined amount of time (maximum of 20 min) in an alkaline liquid (pH of >7.4), reducing the dead space between the coil loops inside the aneurysm (the volume becomes 5–6 times larger than that of a bare platinum coil). Several limitations of first-generation hybrid hydrogel platinum detachable coils (coil stiffness and time restriction for placement) [10, 11] led to the development of second-generation hydrogel coils. These new, supposedly softer coils contain less hydrogel and swell more slowly than hydrogel coils (HydroSoft/HydroFrame and HydroFill; MicroVention, Inc., Aliso Viejo, CA, USA).

The HydroCoil Endovascular Aneurysm Occlusion and Packing Study (HELPS) was performed to evaluate first-generation hydrogel coils in 2011 [12]. The subgroup analysis in the HELPS showed that treatment of irregularly shaped and relatively wide-necked aneurysms with hydrogel coils was associated with significantly lower major and minor recurrence rates than treatment with bare platinum coils during the study period [13]. Since the HELPS, many clinical trials have begun and are currently ongoing, including the new-generation Hydrogel Endovascular Aneurysm Treatment Trial (HEAT) [14], the Hydrogel Coil versus Bare Platinum Coil in Recanalization Imaging Data Registry (HYBRID), and the Japanese HydroSoft Registry (JHSR) [15]. In the present study, the outcome of the use of hydrogel coils suggests that these coils may reduce the incidence of recanalization of embolized aneurysms. Several clinical studies have also shown good results [16–19]. We analyzed the difference in endothelial cell proliferation between bare platinum coils and second-generation hydrogel coils (HydroSoft/HydroFrame) using histochemical and morphological techniques in a swine experimental aneurysm model.

## 2. Materials and Methods

### 2.1. Aneurysm Model

The Animal Care Committee of Juntendo University approved all animal procedures described in this report. Aneurysms were created in 12 adult Landrace-Yorkshire-Duroc swine weighing 30 to 45 kg and ranging in age from 3 to 4 months. The swine were obtained from the National Livestock Breeding Center, Ibaraki Station (Ibaraki, Japan). They were maintained on a 12-hour light/dark cycle with free access to food and water. In both carotid arteries of the 12 swine, 24 experimental aneurysms were surgically created as described previously [20, 21]. The swine were randomly assigned to two groups of six animals each: those subsequently embolized with bare platinum coils and those subsequently embolized with hydrogel coils (HydroSoft/HydroFrame). In brief, after intramuscular injection of ketamine and intubation to maintain ventilation, general anesthesia was performed with muscle relaxation and isoflurane inhalation. Using a sterile technique, the left external jugular vein was exposed and isolated via a midline neck incision. Two venous pouches were harvested, and each was used to create a single end-to-side anastomosis in each of the carotid arteries (vein to artery), with a 2.8 mm neck and 6 mm dome height. The stump of the venous pouch was clipped to allow thrombectomy when the aneurysm had spontaneously thrombosed during the procedure. Cineangiography was performed to ensure that no major bleeding or leakage from the anastomosis occurred before reestablishment of blood flow [22]. This study used the method described by Mitome-Mishima et al. [8], and their wording is partly reproduced in Materials and Methods.

### 2.2. Embolization Procedure

Coil embolization was performed as previously described via the right femoral artery within 12 hours to avoid spontaneous thrombosis [23]. In brief, a 4 Fr sheath was placed in the right femoral artery and connected to a continuous heparinized saline flush. A 4 Fr guiding catheter (JNS Type I; Medikit, Tokyo, Japan) was placed over an angled guidewire (Radifocus Guidewire; Terumo, Tokyo, Japan) and advanced into the proximal right/left common carotid artery. A microcatheter (Excelsior™ SL-10 Microcatheter; Boston Scientific/Target Therapeutics, Fremont, CA, USA) was then inserted coaxially through the guiding catheter (Transend™ Guidewire; Boston Scientific/Target Therapeutics) into the aneurysmal cavity. Cineangiography was performed after injection of 5 mL of iodinated contrast medium (Iopamiron 300; Bayer HealthCare, Leverkusen, Germany). The aneurysms were packed as densely as possible with bare platinum coils (Guglielmi Detachable Coil; Stryker, Tokyo, Japan) or hydrogel coils (HydroFrame 10/HydroSoft 10 Detachable Coil; MicroVention, Inc./Terumo). The packing density was assessed using a previously described classification of angiographic results: class 1, complete obliteration; class 2, residual neck; and class 3, residual aneurysm [24].

### 2.3. Euthanasia Procedures

After assessment of the packing density by cineangiography at 2 or 4 weeks after surgery (*n* = 3 for each group), the swine were euthanized under deep anesthesia with a lethal dose of intravenous pentobarbital (Kyoritsu Seiyaku Corporation, Tokyo, Japan). The aneurysms, parent arteries, and normal arterial tissue (as control specimens) were resected *en bloc* and placed directly into either 4% paraformaldehyde or 2.5% glutaraldehyde phosphate-buffered saline (PBS) solution at 4°C for rapid fixation. The necks of the aneurysms were examined visually and photographed, after which the metallic coil fragments were carefully removed under a dissecting microscope.

### 2.4. Histological Examination

The blocks fixed in paraformaldehyde were embedded in paraffin, and 3 *μ*m coronal sections were cut to allow long-axis examination of the aneurysm neck. For blinded counting of each group, at least three sections from each block were stained with hematoxylin and eosin, azan, or immunohistochemical reagents.

### 2.5. Immunohistochemistry

Sections were deparaffinized and pretreated in an autoclave (121°C, 5 min) in Dako Cytomation Target Retrieval Solution (DakoCytomation, Inc., Copenhagen, Denmark). Slides were left to cool at room temperature (RT) for 1 hour, after which they were rinsed in PBS and incubated in 0.3% hydrogen peroxide diluted with methyl alcohol at RT for 15 min. Blocking was performed with 20% SEA BLOCK™ Blocking Buffer (Pierce Biotechnology, Thermo Fisher Scientific, Waltham, MA, USA) and 1% donkey whole serum diluted in PBS at RT for 30 min. The sections were immunostained overnight at 4°C with antibodies to CD31 (dilution 1 : 100; Abcam, Cambridge, MA, USA), von Willebrand factor (vWF) (dilution 1 : 200; Dako, Glostrup, Denmark), and proliferating cell nuclear antigen (PCNA) (dilution 1 : 100; Dako). The sections were then rinsed with 0.01% Tween 20 in PBS and treated with secondary antibodies (EnVision™ System Labeled Polymer; Dako) at RT for 30 min. Reactions were detected using 0.05% 3,3′-diaminobenzidine tetrahydrochloride (DAB Tablet; Wako Pure Chemical Industries, Osaka, Japan) as a chromogen and 0.015% hydrogen peroxide in PBS. Sections were counterstained with Mayer's hematoxylin, dehydrated, mounted, and examined under a light microscope (E800; Nikon, Tokyo, Japan). Photomicrographs were acquired using a color digital camera affixed to the microscope (Axiocam HRc; Carl Zeiss, Oberkochen, Germany) with AxioVision software version 4.7 (Carl Zeiss).

### 2.6. Immunofluorescence Analysis

Sections were incubated with a primary antibody to CD34 (dilution 1 : 100; Abcam) and vascular endothelial growth factor receptor 2 (VEGFR2) (dilution 1 : 100; Abcam) overnight at 4°C. The sections were then incubated with appropriate secondary antibodies (Alexa Fluor™ Plus 488 donkey anti-rabbit IgG (1 : 25) and Alexa Fluor™ Plus 555 donkey anti-mouse IgG (1 : 25); Thermo Fischer Scientific) for 2 hours at RT. Nuclei were counterstained with ProLong Gold Antifade Reagent with 4′,6-diamidino-2-phenylindole (DAPI) (Cell Signaling Technology, Danvers, MA, USA) and DAPI (Thermo Fischer Scientific). A Leica TCS SP5 confocal microscope (Leica Microsystems, Wetzlar, Germany) and Leica Application Suite X v.3.4.2.18368 image-processing software (Leica Microsystems) were used for image acquisition and analysis.

### 2.7. Transmission Electron Microscopy (TEM)

Aneurysms were processed for TEM after being cut into smaller fragments (1 × 2 mm^2^) and immersed in 2.5% glutaraldehyde solution. These fragments were then washed in PBS, fixed in 2% osmium tetroxide for 2 hours at 4°C, dehydrated with graded concentrations of ethanol, and placed in resin for 4 days at 60°C. Semithin sections were stained with toluidine blue. Ultrathin sections were stained with uranyl acetate and lead citrate, placed on foamer-coated copper grids, and examined under a JEM-1230 electron microscope (JEOL, Tokyo, Japan) at 80 kV.

### 2.8. Cell Counts and Statistical Analysis

**Figure 1:**
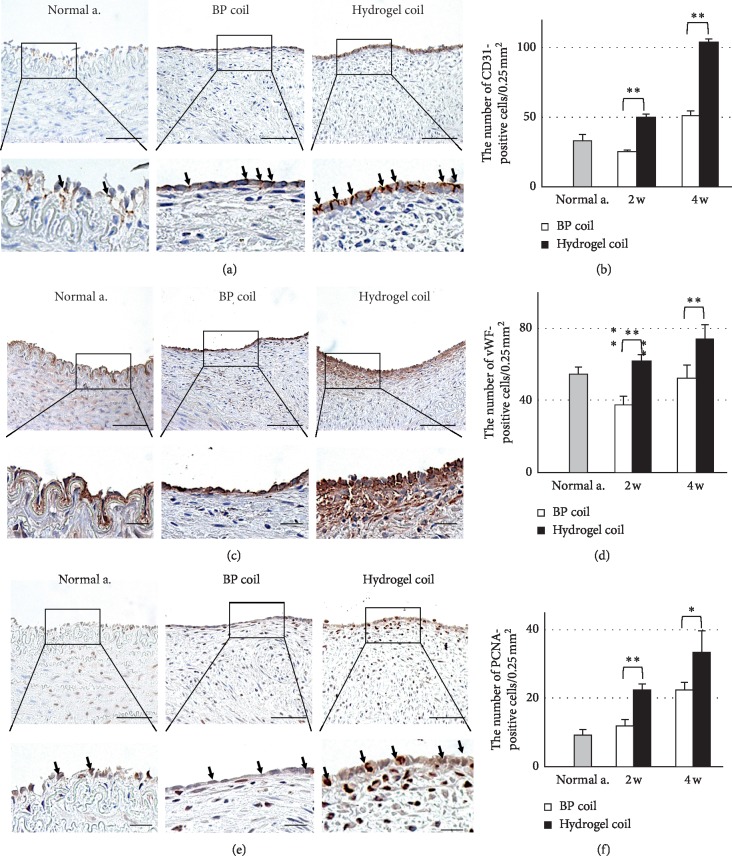
Immunohistochemical analysis of proliferating cells. (a) Photomicrographs of CD31-positive cells 4 weeks after embolization in normal arterial tissue and in the bare platinum (BP) and hydrogel coil groups. Scale bars = 50, 20 *μ*m (scale bar of a larger image is 20 *μ*m). (b) Number of CD31-positive cells. (c) Photomicrographs of vWF-positive cells 4 weeks after embolization. Scale bars = 50, 20 *μ*m (scale bar of a larger image is 20 *μ*m). (d) Number of vWF-positive cells. (e) Photomicrographs of PCNA-positive cells 4 weeks after embolization. Scale bars = 50, 20 *μ*m (scale bar of a larger image is 20 *μ*m). (f) Number of PCNA-positive cells.

In the proliferating tissue, positively immunostained cells were counted in three sections per animal (0.25 mm^2^) (Figures 1(b), 1(d), and 1(f)). Each group comprised six aneurysms from three euthanized swine at three time points. Power estimates were calculated based on *α* = 0.05 and *β* = 0.8 to obtain group sizes sufficiently large to detect effect sizes ranging from 30% to 50% for in vivo models. Statistical significance between two groups was evaluated using Student's unpaired *t*-test, and multiple comparisons were performed using analysis of variance followed by Tukey's honestly significant difference test. Data are expressed as mean ± standard deviation or standard error of the mean. A *p* value <0.05 was considered statistically significant. All analyses were performed using PASW Statistics for Windows, version 18 (SPSS Inc., Chicago, IL, USA).

## 3. Results and Discussion

### 3.1. Angiographic Findings

**Figure 2:**
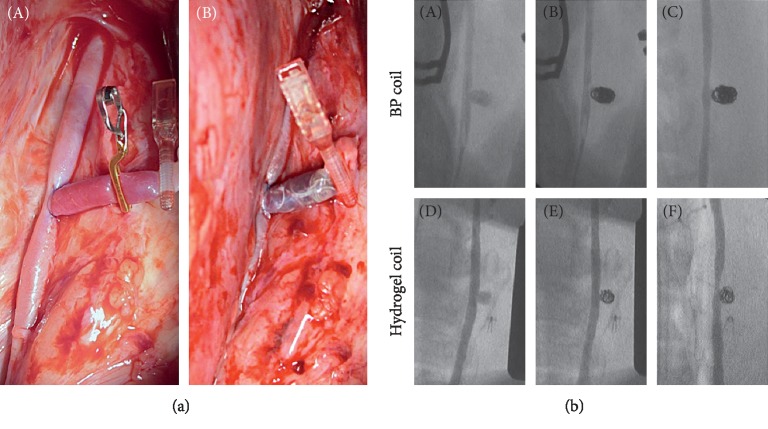
Operative views and angiograms. (a) Intraoperative views of aneurysm (A) before and (B) after embolization. (b) (A, D) Intraoperative angiograms in the bare platinum (BP) and hydrogel coil groups. (B, E) Angiograms obtained immediately after surgery. (C, F) Angiograms obtained before euthanization.

None of the swine died during the experiment, and all were confirmed to have aneurysms in both carotid arteries (Figure 2(a)). Cineangiograms are shown in Figure 2(b). All aneurysms were class 1, with complete obliteration; none of them showed angiographic evidence of coil compaction or recanalization or contrast filling of the neck remnant or dome.

### 3.2. Macroscopic Findings

**Figure 3:**
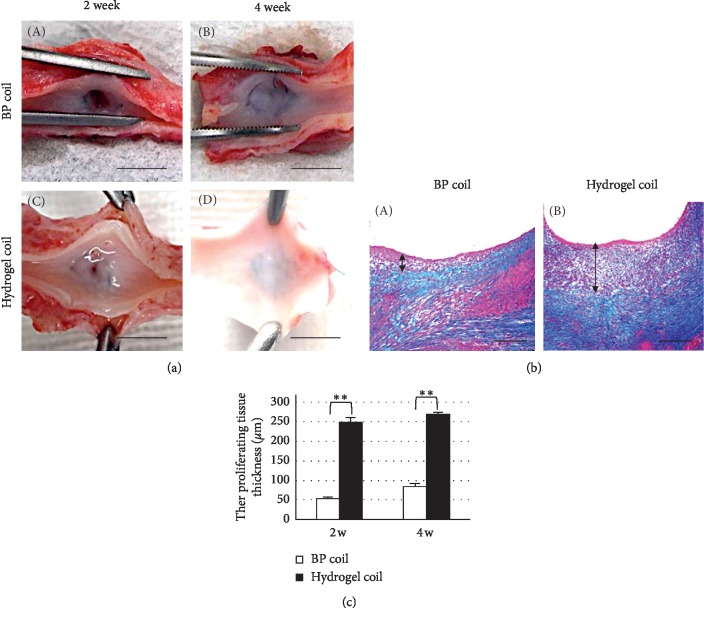
Histological analysis of proliferating tissue. (a) Macroscopic analysis of proliferating tissue 2 and 4 weeks after surgery. Scale bar = 3 mm. (b) Histological analysis of proliferating tissue (azan stain) 4 weeks after surgery in the bare platinum (BP) and hydrogel coil groups. Scale bar = 250 *μ*m. (c) Thickness of proliferating tissue covering the aneurysmal orifices. Data are expressed as mean ± standard error of three swine in each group. ^*∗∗*^*p* < 0.001 between the two groups.

The necks of all aneurysms were covered with a combination of reddish fibrous tissue and a thin membrane of incomplete neointima formation 2 weeks after coil embolization. The fibrous neointimal membranes covering the aneurysm orifice appeared thicker in the hydrogel coil group than in the bare platinum coil group 4 weeks after surgery (Figure 3(a)).

### 3.3. Histological Findings

**Figure 4:**
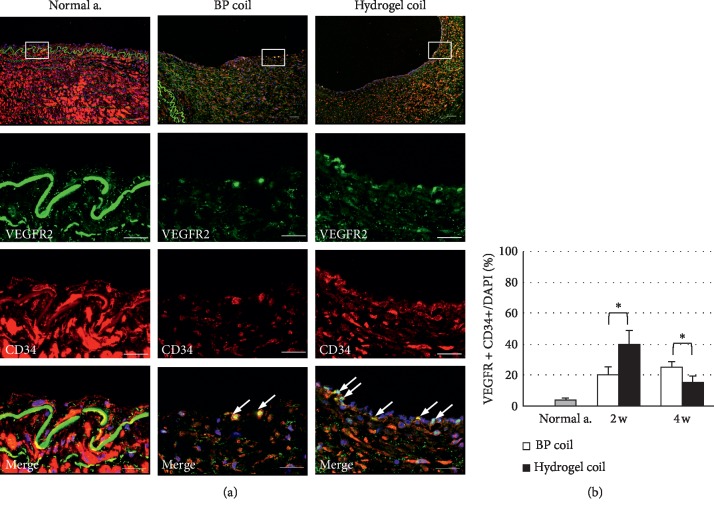
Immunohistochemical analysis of endothelial progenitor cells (EPCs). (a) Double immunofluorescence staining for VEGFR2 (green), CD34 (red), and merged images of proliferating tissue covering the aneurysmal orifice 4 weeks after embolization in normal arterial tissue and in the bare platinum (BP) and hydrogel coil groups. Scale bar = 20 *μ*m. (b) Number of VEGFR2/CD34 double-positive cells. Data are expressed as mean ± standard error of three swine in each group. ^*∗*^*p* < 0.05 and ^*∗∗*^*p* < 0.001 between the two groups.

The aneurysmal sacs were packed with thrombi led by the coils, and lining cell proliferation was seen above the coil mass. The proliferating tissue at the neck of the aneurysm was significantly thicker in the hydrogel coil group in azan-stained sections at 2 and 4 weeks after surgery (*p* < 0.001) (Figures 3(b) and 3(c)). The number of endothelial cells (CD31-positive cells) (Figures 1(a) and 1(b)), the area of the collagen base below the proliferating cells (vWF-positive cells) (Figures 1(c) and 1(d)), and the number of proliferating cells (PCNA-positive cells) (Figures 1(e) and 1(f)) increased over time after embolization. The numbers of CD31- and vWF-positive cells were close to those in normal arterial tissue in the bare platinum coil group and obviously increased in the hydrogel coil group. Additionally, the number of PCNA-positive cells was higher in the hydrogel coil group than in the bare platinum coil group and normal arterial tissue. To determine the origin of proliferating cells, double immunohistochemistry was performed using VEGFR2 and CD34. Some double-positive cells were found in proliferating membranes (Figure 4(a)). The number of VEGFR2/CD34 double-positive cells was rare in normal arterial tissue but gradually increased in the bare platinum coil group. In the hydrogel coil group, the number of these cells increased and peaked at 2 weeks after coil embolization (Figure 4(b)).

### 3.4. TEM Findings

**Figure 5:**
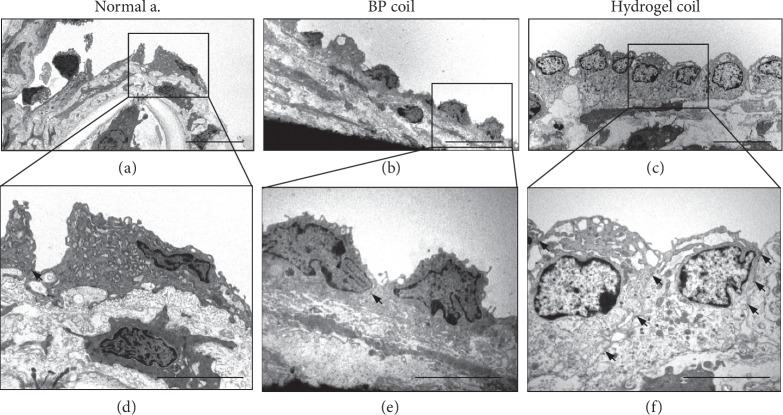
Ultrastructural analysis of proliferating cells. Transmission electron microscopic images of (a, d) normal artery, (b, e) bare platinum (BP) coil group, and (c, f) hydrogel coil group, 4 weeks after embolization. Arrowhead: tight junction. Scale bars: (a–c) 10 *μ*m and (d–f) 5 *μ*m.

Finally, TEM was performed to study the cellular morphology of the proliferating tissue. TEM showed more endothelial-like cells in both the bare platinum coil and hydrogel coil groups than in normal arterial tissue at 4 weeks after surgery. The number of these cells was significantly increased in the hydrogel coil group, and the number of attachments was high for each lining cell. A greater number of tight junctions were present in the hydrogel coil group (Figure 5).

## 4. Discussion

In this experimental study, second-generation hydrogel coils induced a higher rate of endothelial cell proliferation, led to production of more multipotential cells, and had more tight junctions than did bare platinum coils. This indicates that hydrogel coils might contribute to a reduced incidence of aneurysmal recurrence after endovascular therapy.

There are two types of hydrogel coils (first- and second-generation coils), and the two types differ in their structure and role [25]. Confusion may arise when these coils are collectively called “hydrocoils”; instead, they should be referred to as either the HydroCoil Embolic System (first-generation hydrogel coils) or HydroFrame/HydroSoft/HydroFill coils (second-generation hydrogel coils). First-generation hydrogel coils exhibit poor handling, although their effectiveness was demonstrated in a randomized controlled trial [12]. The HELPS revealed an 8.6% lower rate of significant recurrences in patients treated with first-generation hydrogel coils than in those treated with platinum coils [12]. The second-generation hydrogel coils have an inner core of hydrogel and a stretch-resistant filament to improve the packing attenuation, and they expand more slowly with less hydrogel than the first-generation hydrogel coils. Thus, the second-generation hydrogel coils are softer and more easily deployed into the aneurysms than the first-generation hydrogel coils and are equivalent to bare platinum coils in terms of the operators' experience [26]. Randomized controlled multicenter trials have been performed to examine patients with cerebral aneurysms treated by coil embolization with either second-generation hydrogel coils or bare platinum coils [27]. This study demonstrated that second-generation hydrogel coils can be used in a wide spectrum of aneurysms with a risk profile equivalent to that of bare platinum coils. The packing density was significantly higher in aneurysms treated with hydrogel coils [28].

Similar studies have involved the use of second-generation hydrogel coils in a rabbit experimental aneurysm model [26, 29]. The aneurysms were angiographically evaluated for occlusion at 1, 3, 6, and 12 months and analyzed histologically. The second-generation hydrogel coils showed increased rates of stable/progressive occlusion with high levels of volumetric filling, increased neointima formation, and increased thrombus organization. In a canine model of bifurcation aneurysm established by Tsumoto et al. [30], almost all aneurysms remained stable and did not recanalize, and microscopically, most aneurysms showed complete endothelialization and thick neointima formation at the neck surface with no thrombus formation. However, these studies did not involve detailed examinations of the function and ability of the neointima. In the present study, we provided further histological and ultrastructural analysis by immunohistochemistry and TEM to further increase the validity. Moreover, swine experimental aneurysm models are rare because of the high cost and cumbersome procedure. In spite of these limitations, this experimental aneurysm model is highly reproducible with respect to its size, shape, and hemodynamics. Additionally, the size of the arteries is similar to that of humans, allowing us to study the same embolization procedure that would be performed in human patients. Thus, we believe that our findings are applicable to clinical medicine.

We found incomplete neointima formation covering the orifice of the aneurysms in both groups 2 weeks after embolization. The neointima became more stable and functional in each group at 4 weeks after embolization, especially in the hydrogel coil group, which showed complete neointima formation. We also observed thicker fibrous thrombi in the hydrogel coil group (*p* < 0.001). Additionally, the immunohistochemical analysis showed that the hydrogel coil group had a higher rate of endothelial cell proliferation. The number of vWF-positive cells gradually increased in the hydrogel coil group, while no difference was observed in the bare platinum coil group. vWF is produced by endothelial cells and megakaryocytes and is present in blood plasma, subendothelial tissue, and platelets [31]. The presence of vWF-positive endothelial cells implies that proliferating tissue is adherent, making it less likely to detach and hence reducing the likelihood of recanalization. Additionally, the number of PCNA-positive cells was increased in both groups, indicating growth of the neointima over the aneurysm orifice and disruption of intra-aneurysmal flow, further reducing the risk of aneurysmal recanalization. After examining the PCNA-positive cells, we analyzed whether these proliferating tissues have some parts potential for endothelial progenitor cells (EPCs). Although no perfect markers of EPCs are available, Flk1 (i.e., VEGFR2) and CD34 have been previously used to identify some subpopulations of EPCs [32]. EPCs, as an important factor in the process of systemic vascular protection and restoration, have recently been found to play a critical role in endothelial maintenance, the vascular repair process, and postnatal angiogenesis [33]. EPCs are equally involved in repair and reconstruction of the aneurysm neck orifice after embolization of intracranial aneurysms, and an increased number of circulating EPCs can accelerate the repair process of the aneurysm neck endothelial lineage [34]. In this study, the trend of EPC transition suggests that the increase in EPCs in the early stage and repair process occurred earlier in the hydrogel coil group than in the bare platinum coil group. In addition, TEM revealed close adjoining endothelial cells in the hydrogel coil group, and many tight junctions were present. These findings indicate a high rate of endothelial cell proliferation in the hydrogel coil group, which could reduce the incidence of aneurysm recanalization.

This study involving a swine experimental aneurysm model treated with hydrogel coils is the first to report such findings. Although we attempted to immunohistochemically examine collagen, PECAM-1 (CD31), and markers of inflammation (i.e., Iba-1 and Mac-1), we detected no significant difference in the proliferating tissue of both groups. Unfortunately, further histological and ultrastructural analysis is difficult in our institution, and few antibodies are available for swine reactivity. This is one of the limitations of our study.

Although we previously compared Matrix2 with bare platinum coils [8], we only compared hydrogel coils and bare platinum coils in the present study. Because several endovascular approaches are now available for the treatment of intracranial aneurysms, including standard coiling, balloon-assisted coiling, stent-assisted coiling, and flow diversion, Matrix2 coils are no longer frequently used in the clinical setting [35].

The difference in endothelialization and the process of aneurysm healing between ruptured and unruptured coiled aneurysms is important [36]. Moreover, recanalization mostly occurs in ruptured aneurysms [37]. However, no model of ruptured aneurysms is currently available, and this is inferior to postmortem examination of human specimens of post-coil embolization of ruptured intracranial aneurysms. Additionally, recanalization usually occurs several months after treatment, and a 4-week evaluation period is not sufficient to claim that hydrogel coils prevent recanalization. These are additional limitations of this study, and a long-term evaluation would be of interest. We have developed a new swine terminal-type aneurysm model that maintains its patency for 3 months [22], and we intend to use this model for mid- and long-term studies.

## 5. Conclusions

This study has demonstrated that aneurysms embolized with second-generation hydrogel coils build thicker scaffolds for endothelialization and lead to earlier tissue proliferation and maturation than those embolized with bare platinum coils. Second-generation hydrogel coils may be superior to bare platinum coils for preventing aneurysmal recanalization after endovascular treatment of cerebral aneurysms. This is the first study to demonstrate rapid endothelial layer proliferation with high numbers of tight junctions in an experimental swine aneurysm model after embolization with second-generation hydrogel coils.

## Data Availability

The data that support the findings of this study are available from the corresponding author upon reasonable request.
